# Identifying the landscape of developmental toxicity new approach methodologies

**DOI:** 10.1002/bdr2.2075

**Published:** 2022-08-12

**Authors:** Richard A. Becker, Enrica Bianchi, Jessica LaRocca, Mary Sue Marty, Vatsal Mehta

**Affiliations:** ^1^ American Chemistry Council Washington District of Columbia USA; ^2^ Corteva Agriscience Indianapolis Indiana USA; ^3^ Dow Chemical Company Midland Michigan USA; ^4^ The Procter & Gamble Company Mason Ohio USA

**Keywords:** developmental toxicity, literature review, new approach methods, scoping report, Tableau, visualization

## Abstract

**Background:**

The dynamics and complexities of in utero fetal development create significant challenges in transitioning from lab animal‐centric developmental toxicity testing methods to assessment strategies based on new approach methodologies (NAMs). Nevertheless, considerable progress is being made, stimulated by increased research investments and scientific advances, such as induced pluripotent stem cell‐derived models. To help identify developmental toxicity NAMs for toxicity screening and potential funding through the American Chemistry Council's Long‐Range Research Initiative, a systematic literature review was conducted to better understand the current landscape of developmental toxicity NAMs.

**Methods:**

Scoping review tools were used to systematically survey the literature (2010–2021; ~18,000 references identified), results and metadata were then extracted, and a user‐friendly interactive dashboard was created.

**Results:**

The data visualization dashboard, developed using Tableau® software, is provided as a free, open‐access web tool. This dashboard enables straightforward interactive queries and visualizations to identify trends and to distinguish and understand areas or NAMs where research has been most, or least focused.

**Conclusions:**

Herein, we describe the approach and methods used, summarize the benefits and challenges of applying the systematic‐review techniques, and highlight the types of questions and answers for which the dashboard can be used to explore the many different facets of developmental toxicity NAMs.

## 1 INTRODUCTION

The pioneering report, *Toxicity Testing in the 21st Century: A Vision and a Strategy* (NRC, 2007), catalyzed the transformation of chemical hazard identification from a large observational scientific discipline focused on apical adverse effects to a predictive pathway approach based on knowledge of biological processes, computational and bioinformatics methodologies, mechanisms and modes of action (MOAs), key events and key event relationships, and dosimetry. In the years since the publication of the National Academies of Science's report, a number of global initiatives have made considerable progress in developing and applying new approach methodologies (NAMs) (i.e., more human‐relevant in vitro and in silico methods) that reduce, refine, or replace traditional in vivo lab animal toxicity testing for evaluating the safety and potential risks of chemicals and chemical products (EU‐ToxRisk, 2022; Horizon, 2020; LabsExplorer, 2020; OECD, 2022b; SEURAT‐1, 2022; Thomas et al., 2019; USEPA, 2022). These in vitro, in silico, and biological pathway predictive methods are collectively referred to as NAMs. NAMs are broadly defined as “any non‐animal technology, methodology, approach, or combination thereof that can be used to provide information on chemical hazard and risk assessment” (ICCVAM, 2018).

While some may consider NAMs as aspirational, they can no longer be considered only as an optional approach. In Europe, a full ban on animal testing for cosmetics has been in place since March 11, 2013 (EC, 2013a, 2013b). In addition, the chemical safety provisions incorporated into the U.S. Toxic Substances Control Act of 2016 have far‐reaching impacts. This law now requires companies and the U.S. Environmental Protection Agency (EPA), when evaluating toxicity and risks, to use reasonably available existing information, computational toxicology, and bioinformatics, high‐throughput screening methods, and prediction models, the grouping of chemicals into categories, and scientifically valid test methods, including tiered testing and other strategies that reduce or replace the use of vertebrate animals while providing information of equivalent or better scientific quality. In 2019, the EPA Administrator issued a directive (USEPA, 2019) to reduce animal testing and mandated the creation of a work plan focused on actions to accelerate the development, validation, and use of NAMs. Subsequently, EPA issued its first NAMs Work Plan in June 2020 (USEPA, 2020) and an updated NAMs Work Plan in December 2021 (USEPA, 2021b). The objectives of the EPA 2021 NAMs Work Plan, which covers all vertebrate animals, include (a) evaluation of regulatory flexibility for use of NAMs, (b) development of baselines and metrics for assessing progress, (c) developing procedures including integrating in vitro assay and toxicokinetic data as read‐across to establish scientific confidence in NAMs and demonstrate the application to regulatory decisions, (d) development of NAMs that fill critical information gaps, and (e) engagement with stakeholders.

For NAMs to be relied upon to support regulatory and product stewardship decisions requires a priori establishment of sufficient scientific confidence in each method or the integration or combination of methods for different regulatory purposes ranging from prioritizing chemicals or mixtures for further evaluation to hazard prediction using structure–activity relationship (SAR)‐based read‐across when there are developmental and reproductive toxicity (DART) data gaps (Blackburn et al., 2015; Lester, Reis, Laufersweiler, Wu, & Blackburn, 2018; Wu et al., 2013) and ultimately, risk assessment (EC, 2020; Parish et al., 2020; Patlewicz, Simon, Rowlands, Budinsky, & Becker, 2015; Thomas et al., 2019). In June 2020, EPA released its NAMs Work Plan, which focused on meeting its goals to reduce animal testing while “ensuring that the Agency's regulatory, compliance, and enforcement activities, including chemical and pesticide approvals and Agency research, remain fully protective of human health and the environment” (USEPA, 2020). EPA updated this work plan in December 2021 (USEPA, 2021a).

Significant limitations remain in developing and applying NAMs, many of which are particularly challenging. These include, but are not limited to, an inadequate or partial understanding of biological pathways, dose–response and tipping points, incomplete coverage of biological targets and pathways, differences in compound distribution and metabolism compared to in vivo animal models, and relatively simplified assays for inferring integrated physiological responses and chemical compatibility (e.g., nonvolatiles, specific solvents) (see e.g., Becker, 2019; Thomas et al., 2019). While progress is being made in overcoming many of the technical limitations through advancements in technologies, the greater the complexity of the toxicological endpoints NAMs are required to emulate, the greater the challenges.

Historically, the potential for agents to impact mammalian embryo development and maturation has typically been evaluated using in vivo lab animal models, with standardized testing protocols (OECD, 2022a; USEPA, 2021a) that require large numbers of animals. These tests typically evaluate and measure apical effects to evaluate dose responses to characterize and quantify the magnitude and types of adverse effects in offspring exposed in utero. Over the years, many alternative methodologies have been developed to replace either whole or parts of these in vivo developmental toxicity tests or examine MOAs. However, the complexities and dynamism of embryogenesis and histogenesis in utero make efforts to cover the entirety of developmental endpoints exceedingly difficult. In many respects, achieving scientific confidence in developmental toxicity NAMs as a replacement for in vivo testing for the application in regulatory programs and product stewardship represents one of the grand challenges for the regulatory toxicology community. The idea of one‐to‐one replacement—replacing an in vivo lab animal developmental toxicity study with a single NAM—has largely been abandoned due to the recognition that such complex biology cannot be emulated with a single assay. Therefore, attention is increasingly being focused on designing an integrated approach to developmental toxicity testing and assessment using a set of specific NAMs, in a tiered approach, to screen across the breadth of biological space and biological pathways inherent in embryogenesis and histogenesis (Dent et al., 2018). The European Chemicals Agency (ECHA) read‐across assessment framework is currently an approved regulatory approach to support DART data needs (ECHA, 2017); other read‐across tools applicable to DART, such as the Organisation for Economic Co‐operation and Development (OECD) Toolbox and AMBIT, are described by Patlewicz, Helman, Pradeep, and Shah (2017). Read‐across that involves cheminformatics and transcriptomics also provides information about putative MOAs, which will be important in understanding whether any of the proposed alternatives are fit‐for‐purpose (Harrill et al., 2021; Lowe & Williams, 2021; VanderMolen et al., 2020).

The dynamics and complexities of in utero fetal development create significant challenges in transitioning from lab animal‐centric developmental toxicity testing methods to assessment strategies based on NAMs. Despite these challenges, considerable progress is being made (Wu et al., 2013). A decision tree for determining whether a chemical has structural features likely to produce developmental or reproductive toxicity provides a pragmatic approach for prioritization and evaluation. Actualizing the knowledge of key events and key event relationships in Adverse Outcome Pathways (AOPs) into integrated approaches for testing and assessment also holds great promise. However, as discussed by Rajagopal et al. (2022), implementing testing strategies for every AOP to predict developmental toxicity adverse effect outcomes is not necessarily practical or feasible. Consequently, Rajagopal et al. (2022) proposed using a suite of in vitro assays to generate biological activity data aligned with known DART biomarkers to generate points of departure and integrate the results with exposure information to derive screening‐level safety assessments based on margins of exposure. Efforts are also progressing to ensure that in vitro NAMs provide sufficient coverage of the biological processes and mechanistic pathways involved in DART in comparison to intact animals. For example, Janowska‐Sejda, Adeleye, and Currie (2022) described a method to underpin integrated approaches to testing and assessment based on an analysis of candidate DART genes and their molecular initiating events, which in turn can inform the selection of cell systems that can best cover these biological pathways.

An additional challenge for commodity and consumer chemicals is that these chemicals are typically designed and used for performance characteristics, not for efficacy in interacting with defined biological pathway targets, unlike pesticides and pharmaceuticals intentionally designed to act via specific mechanisms within biological organisms. Consequently, commodity and consumer chemicals that are not inherently reactive typically require in vivo testing at much higher doses than pharmaceuticals and pesticides to elicit adverse effects. At such relatively high doses, systemic toxicity is elicited, potentially as a consequence of overwhelming homeostatic pathways. Thus, in biological pathway‐driven integrated approaches for developmental toxicity testing, sorting out mechanism‐specific responses from high‐dose nonspecific responses is needed. Additionally, as virtually all substances can elicit responses under circumstances in which the concentrations in vitro are sufficiently high, criteria in developmental toxicity NAMs must be established for designating a chemical as a true negative. In the in vivo, developmental toxicity test guideline (OECD, 2022a), a limit test of 1,000 mg/kg body weight/day by oral administration can be used. Furthermore, if an in vivo developmental toxicity dose–response study is conducted, the results should “allow for the discrimination between developmental effects occurring in the absence of general toxicity and those which are only expressed at levels that are also toxic to the maternal animal.”

Developmental toxicity NAMs were identified as an area wherein the American Chemistry Council's (ACC) Long‐Range Research Initiative (LRI) could consider devoting additional efforts to advance chemical safety evaluation technologies. The ACC LRI has, over the years, supported research to catalyze the advancement of risk assessment methods, including toxicogenomics, mode of action, —dose–response modeling, physiologically based pharmacokinetic (PBPK) modeling, in vitro to in vivo extrapolation (IVIVE) tools, and computational and in vitro NAMs. To help the LRI program better understand the current status of the range and breadth of developmental toxicity NAMs, we used scoping review tools to gather information. We then organized the results and metadata into a user‐friendly dashboard, utilizing Tableau® software, to enable straightforward interactive queries and visualizations to identify trends and to distinguish and understand areas or NAMs where research has been most, or least, focused. Herein, we describe the approach and methods used, summarize the benefits and challenges of applying the review techniques, and highlight the types of questions and answers this approach and the dashboard can help to identify and address. The developmental NAM dashboard, a nonprofit tool, is freely available for use by the scientific community.

Driven by the ever‐expanding need for evidence‐based research that does not use whole mammalian models, the work presented in this paper utilizes a scoping report, reference prioritization, and data visualization techniques to develop a high‐level overview of the extent and range of NAMs to help inform regulatory decision‐making in regard to the toxicity of stressors on developmental outcomes (DevTox).

## 2 STRUCTURED APPROACH TO SEARCH, SCREEN, SELECT, CATEGORIZE, AND CATALOG STUDY FINDINGS

### 2.1 Step 1: Identifying the research question and relevant studies

#### 2.1.1 1a. General process used

**FIGURE 1:**
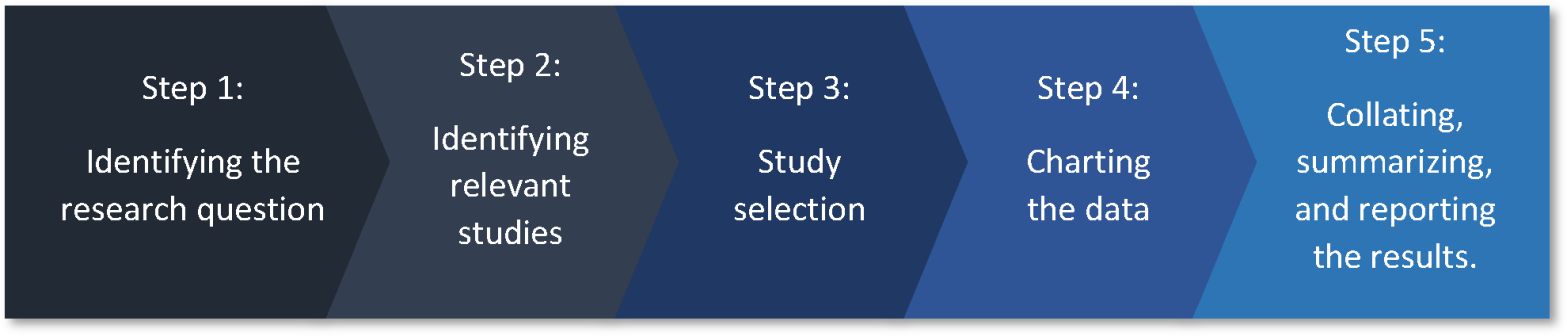
Arksey and O'Malley's five steps to a scoping report (Arksey & O'Malley, 2005)

There are countless ways to collect references and evaluate scientific information relevant to identifying promising research areas to explore for advancing the development and application of DevTox NAMs. A narrative review by one or more subject matter experts was considered one possibility. Instead, we elected to use a predetermined structured method to search, screen, select, categorize, and catalog study findings. The utilization of scoping reports has increased in recent years due to their ability to quickly identify and categorize high‐level available information on a topic. In their 2005 paper, Arksey and O'Malley (2005) outlined the background behind scoping reports and presented their definition of and steps to a scoping report. Furthermore, Levac, Colquhoun, and O'Brien (2010) and Cacchione (2016) expanded upon these methodologies, outlining many additional reasons for conducting a scoping report as well as methodological guidelines. In 2015, these advances led the Joanna Briggs Institute to develop a more advanced set of guidelines surrounding scoping reports (Peters et al., 2015). This in turn led to the development of the Preferred Reporting Items for Systematic Reviews and Meta‐Analyses extension for scoping reviews (PRISMA‐ScR) checklist in 2018 (Tricco et al., 2018). While the details vary between the guidelines presented by these various authors, we focused on the Arksey and O'Malley (2005) outline as it succinctly describes the four major reasons why a scoping report may be conducted, one of which includes identifying research gaps, and lists the five steps involved in conducting a scoping report (Figure 1).

**FIGURE 2:**
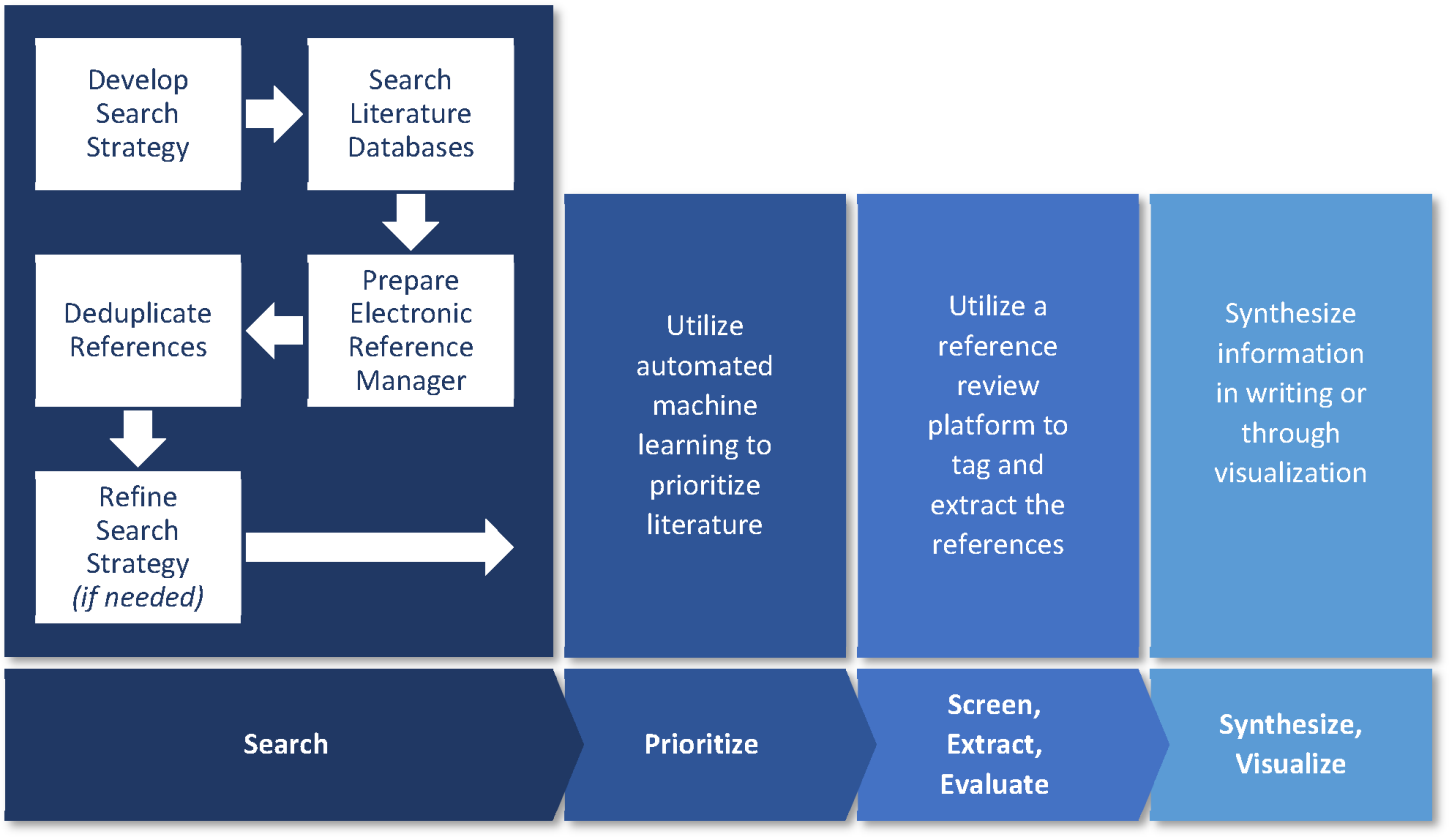
Diagram of literature‐identification process

While the identification and prioritization of the necessary references are critical to the development of the scoping report, an equally important aspect is the dissemination and exploration of the information contained within each reference. A few papers in recent years have utilized some visualization tools in health science scoping reports, such as Chishtie et al. (2020), Goulart et al. (2021), and Jornod et al. (2021), to visualize health outcome information. While these tools are helpful, we chose to use other tools that were more fit‐for‐purpose to project needs. Utilizing litstream™, a screening and extraction software integrated with DoCTER (https://www.icf-docter.com/; last accessed December 17, 2021; https://www.icf.com/technology/litstream; last accessed December 17, 2021), allowed for a seamless transition between prioritization, screening, and extraction steps, as well as transfer to a visualization software, such as Tableau (Cawley et al., 2020; Varghese, Cawley, & Hong, 2018). The general literature‐identification process used is outlined in Figure 2 and described in further detail in the following sections. Figure 2 summarizes the steps, activities, and tools used to implement Arksey and O'Malley's steps 2 through 4.

#### 2.1.2 1b. Search terms

To identify the literature relevant to DevTox NAMs, four “categories” of search terms were developed (specific terms in each category detailed in Table S1). Multiple literature searches were tested in PubMed to determine what combination of search terms resulted in the most useful set of references, with the results of these searches outlined in Table S2.

- “DevTox”—Set of terms related to general health effects and endpoints associated with Developmental Toxicology
- “NAMs”—Set of terms that are associated with already accepted NAMs
- “Models”—Set of terms associated with species and other models used in Developmental Toxicology
- “Pathways”—Set of terms for specific pathways associated with developmental toxicology of known interest

After consideration, a strategy that required terms from each of the four categories was selected as the most likely to be inclusive of the most relevant studies while maximizing the time and budget available.

### 2.2 Steps 2 and 3: Study selection and prioritization methods

Throughout the initial search and screening process, decisions had to be made about what types of references would be prioritized as most beneficial to the project's goals. These decisions were made through group discussions by subject matter experts and information specialists. The final strategy (Table 1) was implemented in PubMed on November 12, 2020; a search update was performed using the same strategy on August 15, 2021. No date limits were applied to the final search strategy.

**TABLE 1:** Detailed literature search strategy^a^

Set	Search strategy for PubMed
DevTox	(((developmental[tiab] OR development[tiab] OR endpoints[tiab] OR endpoint[tiab] OR endpoint determination/trends[mh] OR neurodevelopmental[tiab] OR neurodevelopment[tiab] OR effects[tiab])) AND ((toxicity[tiab] OR toxicities[tiab] OR toxicity[sh] OR toxicant[tiab] OR toxicants[tiab] OR neurotoxicity[tiab])) OR teratogenesis[mh] OR teratogenesis[tiab] OR teratogens[tiab] OR teratogenic[tiab] OR teratology[mh] OR teratology[tiab] OR teratologies[tiab] OR gravidity[mh] OR pregnant[tiab] OR pregnancy[mh] OR pregnancy[tiab] OR gestation[tiab] OR parturition[mh] OR parturition[tiab] OR birth defects[tiab] OR “congenital abnormalities”[mh] OR “congenital abnormalities”[tiab] OR morphology[tiab] OR morphogenesis[mh] OR morphogenesis[tiab] OR dysmorphology[tiab] OR dysmorphologies[tiab] OR dysmorphogenesis[tiab] OR malformation[tiab] OR malformed[tiab] OR “cell differentiation”[mh] OR “cell differentiation”[tiab] OR fetus[mh] OR fetus[tiab] OR fetal[tiab] OR “embryonic structures”[mh] OR embryo[tiab] OR embryonic[tiab] OR conceptus[tiab] OR organogenesis[mh] OR organogenesis[tiab] OR implantation[tiab] OR “embryo implantation”[mh] OR “programmed cell death”[tiab] OR abortion[tiab] OR placenta[mh] OR placenta[tiab] OR “yolk sac”[mh] OR “yolk sac”[tiab] OR ectoderm[mh] OR ectoderm[tiab] OR mesoderm[mh] OR mesoderm[tiab] OR endoderm[mh] OR endoderm[tiab] OR “neural crest”[mh] OR “neural crest”[tiab] OR “neural plate”[mh] OR “neural plate”[tiab] OR notochord[mh] OR notochord[tiab] OR somites[mh] OR somites[tiab] OR “neural tube”[mh] OR “neural tube”[tiab] OR “limb buds”[mh] OR “limb buds”[tiab] OR “limb bud”[tiab] OR larval[tiab] AND develop*[tiab])
NAMs	(assay[tiab] OR assays[tiab] OR “biological assay”[mh] OR pathway[tiab] OR alternative[tiab] OR “Models, Biological”[mh] OR model[tiab] OR approach[tiab] OR assay[tiab] OR “biological assay”[mh] OR profile[tiab] OR predictive[tiab] OR “high‐throughput”[tiab] OR “high throughput”[tiab] OR “high content”[tiab] OR toxicokinetics[tiab] OR transcriptomics[tiab] OR transcriptome[mh] OR HTS[tiab] OR HTTr[tiab] OR “High‐Throughput Screening Assays”[mh] OR “new approach methodologies”[tiab] OR “new approach methods”[tiab] OR NAMs[tiab] OR “Computational Biology/trends”[mh] OR “Animal Testing Alternatives”[mh] OR “Animal Use Alternatives/methods”[mh] OR “Animal Use Alternatives/trends”[mh] OR “alternative approach”[tiab] OR genomic[tiab] OR genomics[tiab] OR “non‐mammalian”[tiab] OR “non‐animal”[tiab] OR “computational toxicology”[tiab] OR “Adverse outcome pathways”[mh] OR “Adverse outcome pathway”[tiab] OR “AOP networks”[tiab] OR biomarkers[mh] OR biomarkers[tiab] OR “in vitro”[tiab] OR “in vitro techniques”[mh] OR “gene expression”[tiab] OR toxicogenomics[tiab] OR in silico[tiab] OR “computer simulation”[mh])
Pathways	(“wnt signaling pathway”[mh] OR “wnt signaling”[tiab] OR “beta catenin”[mh] OR “beta catenin”[tiab] OR JNK[tiab] OR “transforming growth factor beta”[mh] OR “serine receptor”[supplementary concept] OR “protein‐serine–threonine kinases”[mh] OR “protein‐serine–threonine kinases”[tiab] OR “receptors, transforming growth factor beta”[mh] OR “tgf beta”[tiab] OR “serine receptor”[tiab] OR “threonine kinase”[tiab] OR “forkhead box”[tiab] OR “Forkhead Transcription Factors”[mh] OR “O transcription factors”[tiab] OR FOXO[tiab] OR hedgehog[tiab] OR hedgehogs[mh] OR hedgehogs[tiab] OR “patched receptors”[mh] OR “patched receptors”[tiab] OR “patched receptor”[tiab] OR “receptor protein‐tyrosine kinases”[mh] OR “protein‐tyrosine”[tiab] OR “receptor tyrosine kinase”[tiab] OR “monomeric gtp‐binding proteins”[mh] OR “gtp‐binding”[tiab] OR “small g protein”[tiab] OR RAS[tiab] OR Notch[tiab] OR “Notch‐delta”[tiab] OR “delta protein”[supplementary concept] OR “Janus Kinases”[mh] OR “JAK/STAT”[tiab] OR cytoplasm[mh] OR “cytoplasmic tyrosine kinase”[tiab] OR “protein‐tyrosine kinases”[mh] OR “tyrosine kinase”[tiab] OR “nf‐kappa b”[mh] OR “nf‐kappa b”[tiab] OR interleukin‐1[mh] OR interleukin‐1[tiab] OR “toll‐like receptors”[tiab] OR “toll‐like receptors”[mh] OR “toll‐like receptor”[tiab] OR “receptors, cytoplasmic and nuclear”[mh] OR “nuclear hormone receptor”[tiab] OR apoptosis[mh] OR apoptosis[tiab] OR “cell death”[mh] OR “cell death”[tiab] OR “protein tyrosine phosphatases”[mh] OR “protein tyrosine phosphatases”[tiab] OR “phosphotyrosine phosphatase”[tiab] OR RPTPs[tiab] OR “guanylate cyclase”[tiab] OR “guanylate cyclase”[mh] OR “nitric oxide”[tiab] OR “soluble guanylyl cyclase”[mh] OR “guanylyl cyclase”[tiab] OR “G protein‐coupled”[tiab] OR GPCR[tiab] OR “receptors, g protein coupled”[mh] OR “gtp binding”[tiab] OR “g protein”[tiab] OR integrins[mh] OR integrins[tiab] OR integrin[tiab] OR cadherins[tiab] OR cadherins[mh] OR “gap junctions”[tiab] OR “gap junction”[tiab] OR “gap junctions”[mh] OR “ligand‐gated”[tiab] OR cations[mh] OR “cation channels”[tiab] OR “unfolded protein response”[tiab] OR UPR[tiab] OR “unfolded protein response”[mh] OR “replication stress”[tiab] OR (“DNA damage”[tiab] AND (checkpoint[tiab] OR checkpoints[tiab] OR stress[tiab])) OR “cell cycle checkpoints”[mh] OR “cell cycle checkpoints”[tiab] OR “DNA damage”[mh] OR “DNA replication”[mh] OR stemina[tiab] OR signaling[tiab] OR receptor[tiab])
Model	(zebrafish[tiab] OR zebrafish[mh] OR medaka[tiab] OR medakas[tiab] OR xenopus[mh] OR xenopus[tiab] OR “caenorhabditis elegans”[mh] OR “caenorhabditis elegans”[tiab] OR “*C. elegans*”[tiab] OR Drosophila[tiab] OR Drosophila[mh] OR iPS[tiab] OR “pluripotent stem cells”[tiab] OR “pluripotent stem cells”[mh] OR micromass[tiab] OR “human embryonic stem cells”[mh] OR “human embryonic stem cell”[tiab] OR “human embryonic stem cells”[tiab] OR hESC[tiab])

Abbreviations: AOP, adverse outcome pathway; DevTox, developmental outcomes; hESC, human embryonic stem cells; NAMs, new approach methodologies.

^a^ The search strategy was created through an iterative process with input from subject matter experts and information specialists; see text for details.

An iterative approach was utilized wherein project team experts in DevTox NAMs were consulted during each step to determine if the results were appropriate and if NAMs were potentially useful for toxicity assessments before moving to the next step. Prioritization was reflected in the decisions made while selecting the search strategy as well as during the clustering processes. Other decisions are outlined in the sections below and were made to utilize time and budgetary resources as efficiently as possible while accurately identifying a representative sample of studies as outlined in Steps 4 and 5. This iterative approach included discussions of general search term sets as well as the development of specific controlled vocabulary search terms. The terms used were extensive but not exhaustive and likely did not cover the full complexity of the developmental processes. Since the purpose was to focus on the landscape of developmental toxicity NAMs, the search strategy, by design, was tailored to exclude basic research science, since such basic research investigations generally focus on the discovery and characterization of the fundamentals of developmental processes. Although such discovery science uses assays to probe and evaluate developmental processes, we were more interested in assays that more closely fit the definition of NAMs—“a NAM is any technology, methodology, approach, or combination thereof that can be used to provide information on chemical hazard and risk assessment that avoids the use of intact animals” (USEPA, 2021b). During these and subsequent steps, this definition of NAMs served as the touchstone for the subject matter experts and information specialists for discussing and collectively judging relevancy, appropriateness, and utility for toxicity assessments in developing and refining the search and screening strategy. Although such processes and procedures are inherently subjective, the collective knowledge of the subject matter experts was viewed as a necessary step. This search strategy, coupled with the clustering technology (see below) ensured broad coverage of the literature. For example, for stem cells, the approach included consideration of publications containing these terms identified in the topic clustering results: stem, embryonic stem, stem cells, embryonic, human, stem cell, es, human embryonic, mouse, derived, eSCS, es cells, pluripotency, hESCS, pluripotent, HESc, mouse embryonic, and so forth (see Table S2). Thus, even though specific test methods, such as mouse embryonic stem cell assays (Brown, Jacobs, & Fitzpatrick, 2012) were not used as specific search terms, the inclusive search methodology would have captured these at a high level. The details presented in sections 2a through 3b apply to the 2020 initial search. Details for the 2021 update are covered in the Annual Updates section in the Supplemental materials.

#### 2.2.1 2a. Topic extraction

Topic extraction is an automated technique to cluster references based on words they have in common within their titles and abstracts. These clusters of terms were reviewed, and expert engagement was used (see above) to prioritize those that had terms most likely to be relevant to this effort. Some clusters were therefore removed to reduce the number of genomic studies and discovery research into gene structure and function because applied methodologies for NAMs useful in developmental toxicity assessments were the desired topic. Topic extraction results are included in Table S2.

#### 2.2.2 2b. Supervised clustering

Supervised clustering, an automated machine learning technique, was implemented wherein 25 references were identified that were confirmed to be relevant to this effort (referred to as “seeds”). The clustering software identified the studies most like those seeds, represented in an “Ensemble Score,” which is a score (from 1 to 6) indicating how many of the six algorithms in DoCTER included the reference as relevant (Cawley et al., 2020; Varghese et al., 2018). For efficiency, only those references included as relevant in four, five, or all six models were moved to the title and abstract screening step.

#### 2.2.3 3a. Title and abstract screening of literature for relevancy

**FIGURE 3:**
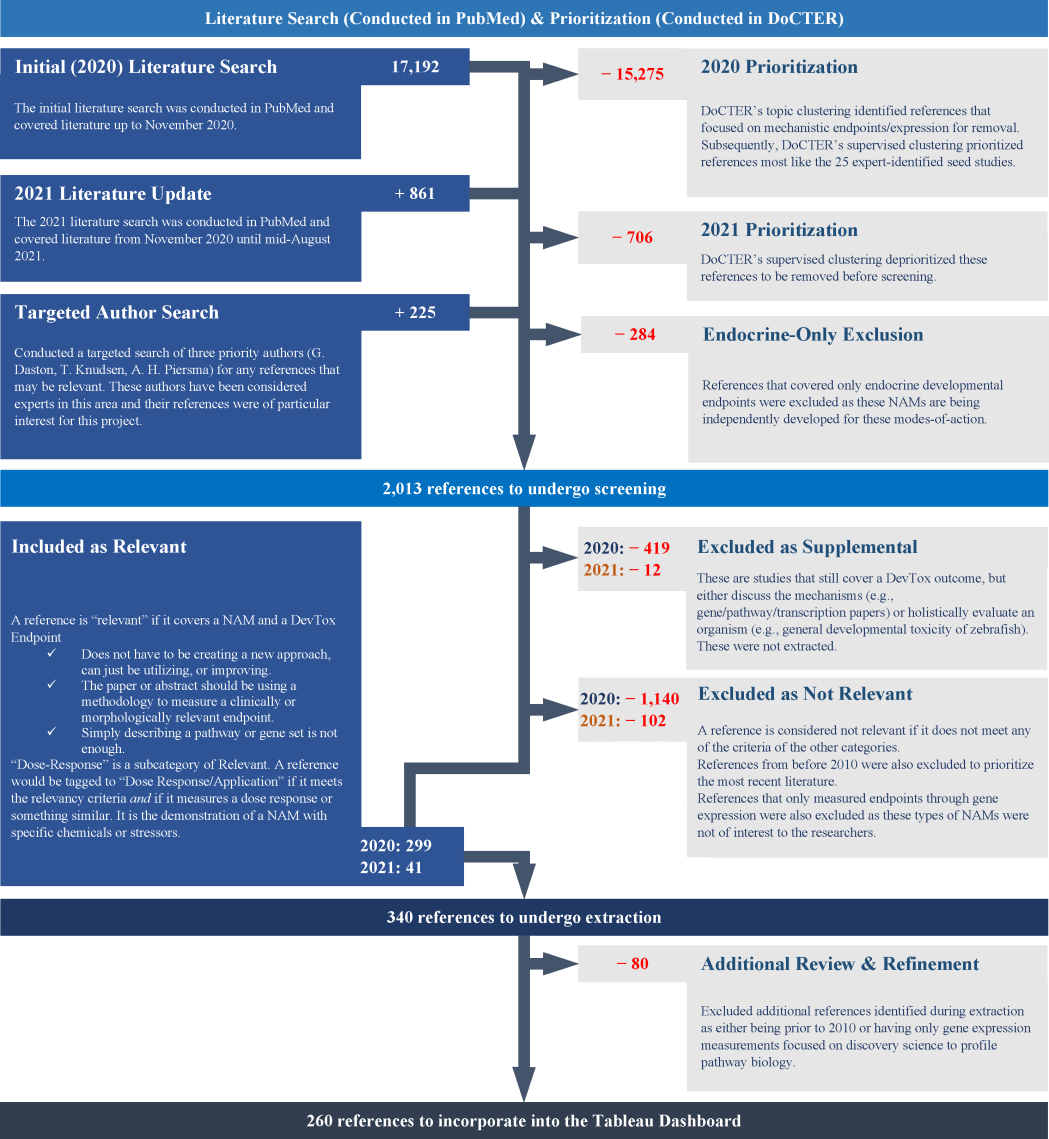
Scoping report process and results

Title and abstract screening were conducted in litstream prior to extracting information so that only relevant references were extracted. Specific criteria were also used to prioritize which references were relevant to this project (Figure 3), keeping in mind the potential future application to developmental toxicity screening. Four different sets of criteria (or tags) were defined to bin the studies. The final results of the title/abstract screening of the prioritized publications can be found in Figure 3.

#### 2.2.4 3b. Extraction of relevant literature

After the screening step was complete, descriptive information was extracted from the titles and abstracts of the relevant studies in litstream. This information was then reviewed by experienced toxicologists, and standardized terminology was used to allow for comparison across references and was imported into a Tableau dashboard (discussed below).

### 2.3 Steps 4 and 5: Charting the relevant literature and results synthesis

Figure 3 summarizes the process used to identify and extract the relevant references; the targeted author search step was added as an extra step to ensure the full set of research publications of these specific experts, who have published in this area, was included. While it is recognized that endocrine‐active substances can also impact development, because significant resources have been devoted over the past 25 years to NAM development and validation activities for estrogen, androgen, steroidogenesis, and thyroid pathways (USEPA, 2017, 2021c), and endocrine NAMs are already being used in regulatory programs (OECD, 2018), we considered this area of developmental toxicity NAMs to already be well developed and elected to exclude references to NAMs for these MOAs. Details on the 2021 literature update are located in the Supplemental materials.

#### 2.3.1 4a. Tableau dashboard

**FIGURE 4:**
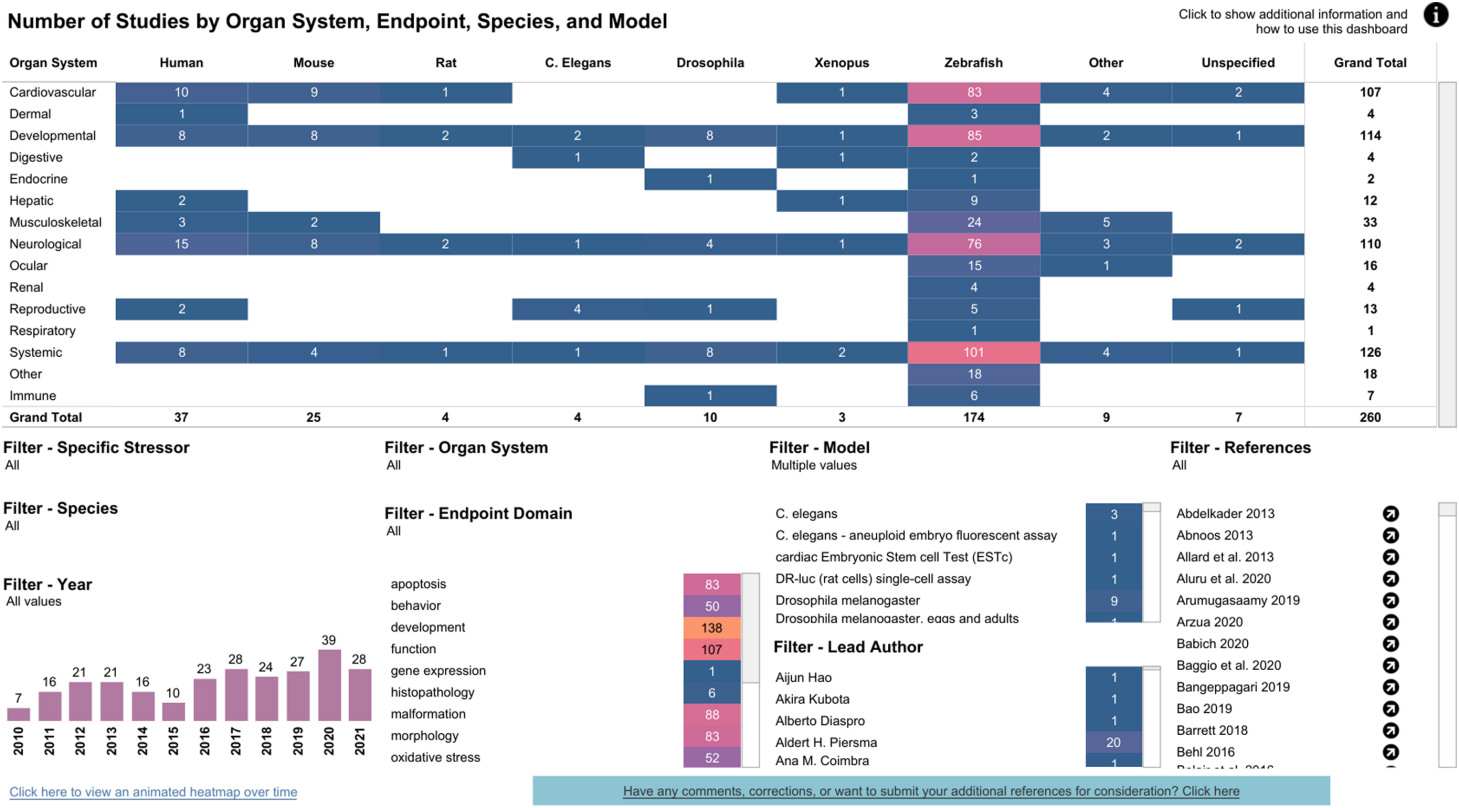
Tableau dashboard

While traditional scoping reports may utilize a narrative approach, the use of interactive Tableau dashboards can be an efficient way to explore the extracted data that can be readily updated. We created an interactive exploratory dashboard (shown in Figure 4; online at https://public.tableau.com/app/profile/acc.vizzes5590/viz/DevelopmentalToxicityNAMsSRResults/Dashboard; last accessed July 8, 2022) so users can easily access, explore, and analyze the DevTox NAMs data. Once data were categorized, distinct counts of studies examining various categories were visualized. Study counts can be viewed in colored “heatmaps” using light‐color shading for smaller counts and darker shading for larger counts. This shading makes it easy to spot where there might be “hotspots” of data—categories with many studies examining the variables (i.e., potentially better‐vetted assays for battery development); or “gaps”—categories with little data or few studies available that may need to be addressed to achieve an effective developmental toxicity screening battery. Visualizing the data in this way also facilitates easy identification of iterative data cleanup needs during data extraction by comparing data across references and making differences more evident. The dashboard can be customized with interactive filters for additional variables of interest to allow further exploration of the available data resulting from systematic categorization. Dashboards are also an excellent way to present final systematic‐review findings to larger audiences.

Additionally, we created a second visualization to support the main dashboard. The supplemental dashboard provides an animation showing the changes that occurred in this research area over time (online at https://public.tableau.com/app/profile/acc.vizzes5590/viz/DevelopmentalToxicityNAMsSRResults-Timeline/Dashboard2; last accessed July 8, 2022).

#### 2.3.2 4b. Components of the DevTox NAMs dashboard

The dashboard is a living resource and contains a Read Me tab to help the user navigate the interactive features and explore the data. Some of the key features are described below.

*Heatmap*: The main heatmap at the top of the page shows numbers of studies by endpoint parameters on the y axis (expandable to multiple columns on the left) and Species on the x axis (across the top). Numbers represent distinct counts of studies, and shading indicates least‐to‐most studies in a light‐to‐dark shaded pattern. A tooltip shows details about examined and measured endpoints activated by hovering over each colored block on the map. With a click or a hover, the user can apply filters, determine counts, and obtain pop‐outs of reference material.

*References*: The references shown in this dashboard are listed in the bottom right of the dashboard by short citation. Hovering over the arrow alongside each reference provides additional reference information as well as clickable PubMed URLs.

*Filters*: A variety of filters are available at the bottom left to filter the dashboard by several variables. Use the drop‐down checkbox filters to include or exclude various values or click on a bar of the “year” bar chart or a row of the “Endpoint Domain” or “Model” filters to filter as well. Once the dashboard has been filtered to the desired references, the information can be downloaded using the download icon in the bottom right of the dashboard and selecting “Data.”

*Customization*: The dashboard is customizable. The user can choose the variables that are featured in the main heatmap, in the filters (and which type of filter), and in the tooltips, allowing a more thorough examination of a topic by eliminating confounding variables.

## 3 DISCUSSION

The developmental toxicity NAMs research dashboard enables interactive queries and visualizations that can be used in a number of ways. For example, a query of the trends in the development of different methods showed the largest number and proportion (>60%) of research publications over the 12‐year period from 2010 to 2021 focused on zebrafish models. This is likely due, in part, to the high genetic homology of the zebrafish with humans, coupled with parallelism in organogenesis and functional mechanisms in a complex organism; approximately 70% of human genes have at least one zebrafish orthologue (Howe et al., 2013). In addition, this could also be because zebrafish embryo assays can be considered nonmammalian NAMs, and, depending on the definition used, as nonanimal models. The United States defines zebrafish embryos as nonlive vertebrates until 72 hr postfertilization (NIH, 2020), and the European Union considers them nonlive vertebrates up to 120 hr (Strähle et al., 2012). In terms of the species used to create NAMs for developmental toxicity evaluation, over the 6‐year period of 2010–2015, the ratio of zebrafish publications to human cell‐based publications was approximately 5:1; for the subsequent period, the ratio changed to approximately 4:1. Human organotypic cellular models were generally lacking in 2010, but over the ensuing 12‐year period, these have expanded considerably such that human‐based development toxicity cellular models now, in 2022, encompass the cardiovascular, reproductive, dermal, hepatic, musculoskeletal, and neurological tissue systems; with the largest number concentrated on neurological and cardiovascular systems.

It has become increasingly clear that the replacement of a traditional in vivo toxicity model with a single alternative assay is unrealistic. Instead, efforts now typically focus on the development of a tiered testing battery composed of complementary, and at times, overlapping to a degree, test systems (discussed in Scialli et al., 2018). Embryonic development and histogenesis are complex dynamic processes for which present knowledge is too incomplete to permit full recapitulation by conceptual and computational modeling. Given this state of knowledge, the dashboard could help identify what a sufficient test battery might encompass. For example in examining the question of what organ systems/cell types would need to be included to cover sufficient biological space, the dashboard is useful in identifying the systems that currently have available assays. Aspirationally, with continued development and refinement, the dashboard may assist in answering the question of what coverage of biological space would be sufficient to allow one to conclude with confidence that a negative result in one or more NAMs is a true negative. Furthermore, the dashboard may be used to identify, for a given organ system or endpoint, a set of NAMs that could be explored to generate comparative data on sensitivity, specificity, and potency to establish sufficient similarity to support the use of one assay over another, to support the use of assays interchangeably, or to select an optimal test set of assays. Similarly, could assays be arrayed in a decision analytic framework matrix to permit rapid segregation of substances that act via selective MOAs (e.g., receptor‐mediated MOAs) from those that act by high‐dose induced effects on homeostasis (e.g., systemic toxicity) (Thomas et al., 2013; Thomas et al., 2019), to enable the latter to be tested in a smaller set of NAMs that could be confidently used to establish a “systemic toxicity” developmental toxicity point of departure for risk‐based decision‐making?

The application of the developmental toxicity NAMs dashboard will depend on the problem formulation and available data. Some potential examples are outlined below.

- The zebrafish model has the highest number of citations across a broad range of endpoints. If establishing a chemical screening program, the dashboard can be used to identify relevant zebrafish studies for evaluating organ/tissue development as well as data gaps. An extensive review may support the conclusion that zebrafish provide a reasonably comprehensive approach to examining development as zebrafish assays have included endpoints evaluating most organ systems (neurological, cardiovascular, systemic, development/malformations, musculoskeletal, reproductive, hepatic, dermal, renal, ocular, immune, and digestive. Note that the respiratory system is represented by gill endpoints). These references also could be mined for information on the domain of applicability and which chemical classes remain to be examined for various developmental endpoints in zebrafish.
- If a chemical is identified as a developmental toxicant in an in vivo study (e.g., cardiac alterations), the dashboard could be used to identify assays that evaluate a similar target organ/system. Once potential assays are identified, in vitro experimental work can confirm whether the selected assay can detect specific developmental toxicity (i.e., treatment‐related cardiac toxicity) at concentrations that do not cause cytotoxicity. If so, the assay may be useful to screen analogues for similar effects on development in an effort to develop chemicals with a more favorable hazard profile.
- Similarly, the dashboard could be used to examine research questions related to relative species sensitivity. If a substance affected neurodevelopmental endpoint(s) in zebrafish, assays in mouse, rat, human, drosophila, *Xenopus*, and *C. elegans* could be reviewed for homologous endpoints to confirm specificity or identify species differences in response. Notably, a positive control chemical would be critical to establish sensitivity across assays for the endpoint of interest. Alternatively, the dashboard can provide a list of human assays that may have relevant endpoints if trying to confirm human sensitivity for a specific assay finding.

For researchers specializing in development of a specific organ/tissue, the dashboard could be used to locate references by tissue/system to determine whether all critical phases of development for the selected organ/tissue are covered by currently available assays (e.g., for neurodevelopment, are their assays that cover neuronal proliferation, migration, differentiation, apoptosis, myelination, etc., or are there gaps that may limit a neurodevelopmental assessment). This approach could identify gaps where targeted assay development would be most valuable. Performing a SAR‐based read‐across continues to be the key methodology to support data needs by extrapolating data from chemicals that have data (source chemical) to the chemical of interest (target chemical) that lacks data (Alexander‐White et al., 2022; Escher et al., 2019). Additional approaches of linking gene expression, AOP, and computational approaches will eventually help reduce the uncertainty in deriving safety thresholds, especially for chemicals that have specific MOAs or a specific target (tissue, receptor). IVIVE has become an important tool for using NAMs‐derived effects data in human health risk‐based prioritization and screening. However, there are many challenges involved in conducting IVIVE from cell‐based assays/in vitro organotypic human models, and detailed step‐by‐step IVIVE procedures for developmental toxicity NAMs are currently lacking. Conceptually, these IVIVE procedures will need to include methods to account for differential partitioning and availability of chemicals in in vitro toxicity assay systems to translate nominal in vitro concentrations to site‐of‐action concentrations. They will also need to include methods to make PBPK predictions during pregnancy in humans and in laboratory animals as well as calculate internal doses at target sites in the maternal and developing organisms. The reason for including in vivo laboratory animal PBPK models is to enable point of departure comparisons of in vitro and in vivo empirical data sets, since empirical human data are not available for the vast majority of substances. Initial IVIVE research in this area has recently been communicated (Chang et al., 2021).

Although it is widely known that the zebrafish NAM for developmental toxicity is one of, if not the most studied alternative methods, the data gathered in the current study confirms the frequent use of this method. Using the dashboard to focus specifically on the zebrafish studies shows that the model is capable of evaluating a breadth of organ systems. This may be an important consideration in designing NAMs‐based developmental toxicity integrated approach to testing and technology which covers a large range of organ systems. Although not discussed here in detail, we have begun to use the dashboard to explore the types of chemicals that have been evaluated in the zebrafish developmental toxicity NAM models to better understand the domain of applicability that has been empirically established to date. From the perspective of our research program, we are interested in better understanding the set of true known positives and the set of truly known negatives (e.g., Jarque et al. (2020)) that have been, or can be, used to establish the sensitivity and specificity of the assays (and different assay endpoints) with an eye toward perhaps funding research to fill in knowledge gaps to improve scientific confidence when using these assays as alternatives to the traditional in vivo test systems.

In addition, we could use the dashboard to consider the types and ranges of modes or mechanisms of action that have been evaluated to date, to help elucidate response patterns exhibited by specific modes/mechanisms compared to response patterns exhibited by chemicals acting via systemic toxicity and not specific modes or mechanisms, since a large number of consumer and commodity chemicals elicit effects as a consequence of overwhelming homeostatic pathways (e.g., high‐dose nonspecific responses). Such an evaluation may reveal a need to support further research to expand the zebrafish assay empirical data set to improve interpretation of the nature and magnitude of endpoint responses and the types of dose–response profiles produced under in vitro exposure conditions that mimic systemic toxicity. Another use of the dashboard could be to support AOP development as specific mechanisms of action are characterized.

Although not the exact focus of this exercise, our evaluation of the dashboard led us to ask, given that the zebrafish models encompass the majority of developmental toxicity NAMs research publications gathered in this activity, how would one calculate a human‐equivalent dose from a point of departure derived from a zebrafish experiment? For human health risk‐based decision‐making, IVIVE methodologies to convert in vitro results to human‐equivalent doses have been instrumental for comparing in vitro results to human exposure predictions to enable risk‐based prioritization and decision‐making. Theoretically, an IVIVE approach could be developed by linking a PBPK model of the zebrafish embryo in vitro system to a human in the utero PBPK model.

However, even if dose extrapolation is possible from the in vitro zebrafish developmental toxicity NAM to derive a human‐equivalent dose, from a species extrapolation perspective, is the developmental ontology in the zebrafish model sufficiently comparable to the mammalian developmental ontology to confidently support cross‐species extrapolation? This question also arose from discussions stimulated by review and querying of the dashboard. In addition to empirical studies (Jarque et al., 2020), research highly relevant to this question is focused on constructing a developmental ontology to describe the pathways and processes critical to embryonic development in a manner that would permit analyzing and linking chemically induced responses in specific pathways and physiological processes to adverse outcomes (Heusinkveld et al., 2021; Staal, Pennings, Hessel, & Piersma, 2017).

## 4 CONCLUSIONS

In the past, activities focused on identifying potential research program priorities for the ACC LRI typically relied on literature reviews or summaries prepared by one or more experts. While there is considerable utility in this approach, it can also be impacted by conscious or unconscious bias in selecting and analyzing existing knowledge. Based on our familiarity with how systematic‐review methods have been applied in toxicity risk evaluations, once we decided to examine the field of developmental toxicity NAMs to better understand the trajectory and current status of assay development, we used a set of systematic‐review technologies to develop a scoping report, reference prioritization, and data visualization techniques to create the developmental toxicity NAMs research dashboard. The dashboard provides an interactive tool that can be queried in a variety of fashions to provide both overviews and details of many different facets of developmental toxicity NAMs.

The dashboard is not a static work product. We intend to continue to update the literature search, reference prioritization procedures, and dashboard fields through at least December 2023. By making the dashboard publicly available, we hope others will explore its use for their purposes. While the systematic literature review methodologies that were employed to develop the dashboard serve to increase transparency, promote objectivity, and enhance reproducibility, the processes and procedures are still a human enterprise and as such are imperfect. Thus, the dashboard needs to be viewed as a living resource, which can evolve and improve over time. To ensure that the dashboard is representative of current developmental toxicity NAMs research, a literature update will be conducted every year at least through 2023 (see Supplemental materials). Users of the dashboard are also encouraged, via an online comment form contained in the dashboard, to provide comments and corrections, suggest additional search terms, provide additional data, and so forth for consideration in the dashboard updates. We are hopeful that the broader developmental toxicity NAM community will see the utility of this approach, evaluate the dashboard, and provide comments and suggestions that will improve this tool over the next few years. Importantly, we anticipate and encourage suggestions to improve the coverage of the complexity of developmental processes, and suggestions to add NAMs and biological pathways important for developmental toxicity that might have been missed in developing this initial version of the dashboard.

## FUNDING INFORMATION

Support for this research was provided by the American Chemistry Council's Long‐Range Research Initiative via contract work assignments with International Coaching Federation (ICF).

## CONFLICT OF INTEREST

Richard A. Becker is employed by ACC, a trade association of U.S. chemical manufacturers. Enrica Bianchi, Jessica LaRocca, M. Sue Marty, and Vatsal Mehta are employed by companies that produce chemicals and or consumer products.

## Supporting information


**Table S1** Search Term Combination Results
**Table S2**. Topic Clustering Results

## Data Availability

The data that support the findings of this study are available from the corresponding author upon reasonable request.

## References

[bdr22075-bib-0001] Alexander‐White, C. , Bury, D. , Cronin, M. , Dent, M. , Hack, E. , Hewitt, N. J. , … Europe, C. (2022). A 10‐step framework for use of read‐across (RAX) in next generation risk assessment (NGRA) for cosmetics safety assessment. Regulatory Toxicology and Pharmacology, 129, 105094. 10.1016/j.yrtph.2021.105094 34990780

[bdr22075-bib-0002] Arksey, H. , & O'Malley, L. (2005). Scoping studies: Towards a methodological framework. International Journal of Social Research Methodology, 8(1), 19–32. 10.1080/1364557032000119616

[bdr22075-bib-0003] Becker, R. A. (2019). Transforming regulatory safety evaluations using new approach methodologies: A perspective of an industrial toxicologist. Current Opinion in Toxicology, 15, 93–98. 10.1016/j.cotox.2019.07.002

[bdr22075-bib-0004] Blackburn, K. , Daston, G. , Fisher, J. , Lester, C. , Naciff, J. M. , Rufer, E. S. , … Woeller, K. (2015). A strategy for safety assessment of chemicals with data gaps for developmental and/or reproductive toxicity. Regulatory Toxicology and Pharmacology, 72(2), 202–215. 10.1016/j.yrtph.2015.04.006 25910676

[bdr22075-bib-0005] Brown, E. S. , Jacobs, A. , & Fitzpatrick, S. (2012). Reproductive and developmental toxicity testing: From in vivo to in vitro. ALTEX, 29(3), 333–339. 10.14573/altex.2012.3.333 23019687

[bdr22075-bib-0006] Cacchione, P. Z. (2016). The evolving methodology of scoping reviews. Clinical Nursing Research, 25(2), 115–119. 10.1177/1054773816637493 26976609

[bdr22075-bib-0007] Cawley, M. , Beardslee, R. , Beverly, B. , Hotchkiss, A. , Kirrane, E. , Sams, R. , … Cowden, J. (2020). Novel text analytics approach to identify relevant literature for human health risk assessments: A pilot study with health effects of in utero exposures. Environment International, 134, 105228. doi:10.1016/j.envint.2019.105228 31711016 PMC10029921

[bdr22075-bib-0008] Chang, X. , Palmer, J. , Donley, E. , Allen, D. , Casey, W. M. , & Kleinstreuer, N. (2021). NICEATM Abstract: ASCCT 2021 Annual Meeting: In vitro to in vivo extrapolation for developmental toxicity potency of selected Tox21 chemicals . https://ntp.niehs.nih.gov/iccvam/meetings/ascct-2021/chang_ascct2021_508.pdf.

[bdr22075-bib-0009] Chishtie, J. A. , Marchand, J. S. , Turcotte, L. A. , Bielska, I. A. , Babineau, J. , Cepoiu‐Martin, M. , … Jaglal, S. (2020). Visual analytic tools and techniques in population health and health services research: Scoping review. Journal of Medical Internet Research, 22(12), e17892. 10.2196/17892 33270029 PMC7716797

[bdr22075-bib-0010] Dent, M. , Amaral, R. T. , Da Silva, P. A. , Ansell, J. , Boisleve, F. , Hatao, M. , … Kojima, H. (2018). Principles underpinning the use of new methodologies in the risk assessment of cosmetic ingredients. ComputationalToxicology, 7, 20–26. 10.1016/j.comtox.2018.06.001

[bdr22075-bib-0011] Escher, S. E. , Kamp, H. , Bennekou, S. H. , Bitsch, A. , Fisher, C. , Graepel, R. , … Kroese, D. (2019). Towards grouping concepts based on new approach methodologies in chemical hazard assessment: The read‐across approach of the EU‐ToxRisk project. Archives of Toxicology, 93(12), 3643–3667. 10.1007/s00204-019-02591-7 31781791

[bdr22075-bib-0012] European Chemicals Agency (ECHA) . (2017). Read‐across assessment framework (RAAF) . https://echa.europa.eu/documents/10162/13628/raaf_en.pdf/614e5d61-891d-4154-8a47-87efebd1851a#:~:text=This%20is%20called%20read%2Dacross,(source%20substance(s))

[bdr22075-bib-0013] European Commission (EC) . (2013a). Full EU ban on animal testing for cosmetics enters into force . https://ec.europa.eu/commission/presscorner/detail/en/IP_13_210

[bdr22075-bib-0014] European Commission (EC) . (2013b). Ban on animal testing . https://ec.europa.eu/growth/sectors/cosmetics/ban-animal-testing_en#:~:text=The%20marketing%20ban%20applies%20since,of%20alternative%20non%2Danimal%20tests

[bdr22075-bib-0015] European Commission (EC) . (2020). *Commission staff working document: Progress report on the assessment and management of combined exposures to multiple chemicals (chemical mixtures) and associated risks*. Accompanying the document: Communication from the Commission to the European Parliament, the Council, the European Economic and Social Committee and the Committee of the Regions chemicals strategy for sustainability towards a toxic‐free environment Brussels, Belgium. SWD(2020) 250 final. https://ec.europa.eu/environment/pdf/chemicals/2020/10/SWD_mixtures.pdf

[bdr22075-bib-0016] EU‐ToxRisk . (2022). EU‐ToxRisk . http://www.eu-toxrisk.eu/

[bdr22075-bib-0017] Goulart, C. M. , Purewal, A. , Nakhuda, H. , Ampadu, A. , Giancola, A. , Kortenaar, J. L. , & Bassani, D. G. (2021). Tools for measuring gender equality and women's empowerment (GEWE) indicators in humanitarian settings. Conflict and Health, 15(1), 39. doi:10.1186/s13031-021-00373-6 34001201 PMC8127307

[bdr22075-bib-0018] Harrill, J. A. , Viant, M. R. , Yauk, C. L. , Sachana, M. , Gant, T. W. , Auerbach, S. S. , … Whelan, M. (2021). Progress towards an OECD reporting framework for transcriptomics and metabolomics in regulatory toxicology. Regulatory Toxicology and Pharmacology, 125, 105020. 10.1016/j.yrtph.2021.105020 34333066 PMC8808338

[bdr22075-bib-0019] Heusinkveld, H. J. , Staal, Y. C. M. , Baker, N. C. , Daston, G. , Knudsen, T. B. , & Piersma, A. (2021). An ontology for developmental processes and toxicities of neural tube closure. Reproductive Toxicology, 99, 160–167. 10.1016/j.reprotox.2020.09.002 32926990 PMC10083840

[bdr22075-bib-0020] Horizon 2020 . (2020). What is Horizon 2020? https://ec.europa.eu/programmes/horizon2020/en/what-horizon-2020

[bdr22075-bib-0021] Howe, K. , Clark, M. D. , Torroja, C. F. , Torrance, J. , Berthelot, C. , Muffato, M. , … Stemple, D. L. (2013). The zebrafish reference genome sequence and its relationship to the human genome. Nature, 496(7446), 498–503. 10.1038/nature12111 23594743 PMC3703927

[bdr22075-bib-0022] Interagency Coordinating Committee on the Validation of Alternative Methods (ICCVAM) . (2018). A strategic roadmap for establishing new approaches to evaluate the safety of chemicals and medical products in the United States . https://ntp.niehs.nih.gov/go/iccvam-rdmp

[bdr22075-bib-0023] Janowska‐Sejda, E. I. , Adeleye, Y. , & Currie, R. A. (2022). Exploration of the DARTable genome – A resource enabling data‐driven NAMs for developmental and reproductive toxicity prediction. Frontiers in Toxicology, 3, 806311. 10.3389/ftox.2021.806311 35295108 PMC8915813

[bdr22075-bib-0024] Jarque, S. , Rubio‐Brotons, M. , Ibarra, J. , Ordoñez, V. , Dyballa, S. , Miñana, R. , & Terriente, J. (2020). Morphometric analysis of developing zebrafish embryos allows predicting teratogenicity modes of action in higher vertebrates. Reproductive Toxicology, 96, 337–348. doi:10.1016/j.reprotox.2020.08.004 32822784

[bdr22075-bib-0025] Jornod, F. , Jaylet, T. , Blaha, L. , Sarigiannis, D. , Tamisier, L. , & Audouze, K. (2021). AOP‐helpFinder webserver: A tool for comprehensive analysis of the literature to support adverse outcome pathways development. Bioinformatics, 38(4), 1173–1175. 10.1093/bioinformatics/btab750 34718414 PMC8796376

[bdr22075-bib-0026] LabsExplorer . (2020). Horizon Europe: Introducing the EU's new framework programme . https://www.labsexplorer.com/c/horizon-europe-introducing-the-eu-s-new-framework-programme_211#:~:text=The%209th%20Framework%20Programme%2C%20also,we%20have%20got%20you%20covered

[bdr22075-bib-0027] Lester, C. , Reis, A. , Laufersweiler, M. , Wu, S. , & Blackburn, K. (2018). Structure activity relationship (SAR) toxicological assessments: The role of expert judgment. Regulatory Toxicology and Pharmacology, 92, 390–406. 10.1016/j.yrtph.2017.12.026 29305951

[bdr22075-bib-0028] Levac, D. , Colquhoun, H. , & O'Brien, K. K. (2010). Scoping studies: Advancing the methodology. Implementation Science, 5(1), 69. 10.1186/1748-5908-5-69 20854677 PMC2954944

[bdr22075-bib-0029] Lowe, C. N. , & Williams, A. J. (2021). Enabling high‐throughput searches for multiple chemical data using the U.S.‐EPA CompTox Chemicals Dashboard. Journal of Chemical Information and Modeling, 61(2), 565–570. 10.1021/acs.jcim.0c01273 33481596 PMC8630643

[bdr22075-bib-0030] National Institute of Health (NIH) . (2020). Guidelines for use of zebrafish in the NIH Intramural Research Program https://oacu.oir.nih.gov/system/files/media/file/2021-02/b17_zebrafish.pdf

[bdr22075-bib-0031] National Research Council (NRC) . (2007). Toxicity testing in the 21st century: A vision and a strategy. Washington, DC: The National Academies Press.

[bdr22075-bib-0032] Organisation for Economic Co‐operation and Development (OECD) . (2018). *Revised guidance document 150 on standard test guidelines for evaluating chemicals for endocrine disruption*. OECD Series on Testing and Assessment. 10.1787/9789264304741-en.

[bdr22075-bib-0033] Organisation for Economic Co‐operation and Development (OECD) . (2022a). OECD guidelines for the testing of chemicals, section 4: Health effects . http://www.oecd‐ilibrary.org/environment/oecd‐guidelines‐for‐the‐testing‐of‐chemicals‐section‐4‐health‐effects_20745788?_ga=2.188897443.903729400.1638471164‐2049234618.1625759743&_gl=1*uhj4kn*_ga*MjA0OTIzNDYxOC4xNjI1NzU5NzQz*_ga_F7KSNTXTRX*MTYzODQ3MTE2My4xLjEuMTYzODQ3MTUwNS4w

[bdr22075-bib-0034] Organisation for Economic Co‐operation and Development (OECD) (2022b). Omics technologies in chemical testing . https://www.oecd.org/chemicalsafety/testing/omics.htm

[bdr22075-bib-0035] Parish, S. T. , Aschner, M. , Casey, W. , Corvaro, M. , Embry, M. R. , Fitzpatrick, S. , … Boobis, A. (2020). An evaluation framework for new approach methodologies (NAMs) for human health safety assessment. Regulatory Toxicology and Pharmacology, 112, 104592. doi:10.1016/j.yrtph.2020.104592 32017962

[bdr22075-bib-0036] Patlewicz, G. , Helman, G. , Pradeep, P. , & Shah, I. (2017). Navigating through the minefield of read‐across tools: A review of in silico tools for grouping. Computational Toxicology, 3, 1–18. https://www.sciencedirect.com/science/article/abs/pii/S2468111317300282?via%3Dihub 30221211 10.1016/j.comtox.2017.05.003 PMC6134855

[bdr22075-bib-0037] Patlewicz, G. , Simon, T. W. , Rowlands, J. C. , Budinsky, R. A. , & Becker, R. A. (2015). Proposing a scientific confidence framework to help support the application of adverse outcome pathways for regulatory purposes. Regulatory Toxicology and Pharmacology, 71(3), 463–477. 10.1016/j.yrtph.2015.02.011 25707856

[bdr22075-bib-0038] Peters, M. , Godfrey, C. , McInerney, P. , Soares, C. , Khalil, H. , & Parker, D. (2015). Methodology for JBI scoping reviews, in The Joanna Briggs Institute Reviewers Manual 2015 (pp. 3–24). Australia: Joanna Briggs Institute.

[bdr22075-bib-0039] Rajagopal, R. , Baltazar, M. T. , Carmichael, P. L. , Dent, M. P. , Head, J. , Li, H. , … Kukic, P. (2022). Beyond AOPs: A mechanistic evaluation of NAMs in DART testing. Frontiers in Toxicology, 4, 838466. 10.3389/ftox.2022.838466 35295212 PMC8915803

[bdr22075-bib-0040] Scialli, A. R. , Daston, G. , Chen, C. , Coder, P. S. , Euling, S. Y. , Foreman, J. , … Thompson, K. E. (2018). Rethinking developmental toxicity testing: Evolution or revolution? Birth Defects Research, 110(10), 840–850. doi:10.1002/bdr2.1212 29436169 PMC6624839

[bdr22075-bib-0041] SEURAT‐1 . (2022). SEURAT‐1 . http://www.seurat-1.eu/

[bdr22075-bib-0042] Staal, Y. C. M. , Pennings, J. L. A. , Hessel, E. V. S. , & Piersma, A. H. (2017). Advanced toxicological risk assessment by implementation of ontologies operationalized in computational models. Applied In Vitro Toxicology, 3(4), 325–332. 10.1089/aivt.2017.0019

[bdr22075-bib-0043] Strähle, U. , Scholz, S. , Geisler, R. , Greiner, P. , Hollert, H. , Rastegar, S. , … Braunbeck, T. (2012). Zebrafish embryos as an alternative to animal experiments—A commentary on the definition of the onset of protected life stages in animal welfare regulations. Reproductive Toxicology, 33(2), 128–132. 10.1016/j.reprotox.2011.06.121 21726626

[bdr22075-bib-0044] Thomas, R. S. , Bahadori, T. , Buckley, T. J. , Cowden, J. , Deisenroth, C. , Dionisio, K. L. , … Williams, A. J. (2019). The next generation blueprint of computational toxicology at the U.S. Environmental Protection Agency. Toxicological Sciences, 169(2), 317–332. 10.1093/toxsci/kfz058 30835285 PMC6542711

[bdr22075-bib-0045] Thomas, R. S. , Philbert, M. A. , Auerbach, S. S. , Wetmore, B. A. , Devito, M. J. , Cote, I. , … Nong, A. (2013). Incorporating new technologies into toxicity testing and risk assessment: Moving from 21st century vision to a data‐driven framework. Toxicological Sciences, 136(1), 4–18. 10.1093/toxsci/kft178 23958734 PMC3829570

[bdr22075-bib-0046] Tricco, A. C. , Lillie, E. , Zarin, W. , O'Brien, K. K. , Colquhoun, H. , Levac, D. , … Straus, S. E. (2018). PRISMA extension for scoping reviews (PRISMA‐ScR): Checklist and explanation. Annals of Internal Medicine, 169(7), 467–473. 10.7326/m18-0850 30178033

[bdr22075-bib-0047] U.S. Environmental Protection Agency (USEPA) . (2017). Continuing development of alternative high‐throughput screens to detect endocrine disruption, focusing on androgen receptor, steroidogenesis, and thyroid pathways. Washington, DC: Endocrine Disruptor Screening Program. https://www.epa.gov/sites/default/files/2017-09/documents/2017_edsp_white_paper_public.pdf

[bdr22075-bib-0048] U.S. Environmental Protection Agency (USEPA) . (2019). Administrator memo prioritizing efforts to reduce animal testing , September 10, 2019. https://www.epa.gov/research/administrator-memo-prioritizing-efforts-reduce-animal-testing-september-10-2019

[bdr22075-bib-0049] U.S. Environmental Protection Agency (USEPA) . (2020). *New approach methods work plan: Reducing use of animals in chemical testing*. Washington, DC: U.S. Environmental Protection Agency, Office of Research and Development, Office of Chemical Safety and Pollution Prevention. EPA 615B20001/June 2020. https://www.epa.gov/sites/default/files/2020‐06/documents/epa_nam_work_plan.pdf

[bdr22075-bib-0050] U.S. Environmental Protection Agency (USEPA) . (2021a). Series 870 – Health effects test guidelines . Washington, DC. https://www.epa.gov/test-guidelines-pesticides-and-toxic-substances/series-870-health-effects-test-guidelines

[bdr22075-bib-0051] U.S. Environmental Protection Agency (USEPA) . (2021b). *New approach methods work plan*. Washington, DC: U.S. Environmental Protection Agency, Office of Research and Development, Office of Chemical Safety and Pollution Prevention. EPA 600/X‐21/209. https://www.epa.gov/system/files/documents/2021-11/nams-work-plan_11_15_21_508-tagged.pdf

[bdr22075-bib-0052] U.S. Environmental Protection Agency (USEPA) . (2021c). *Endocrine Disruptor Screening Program (EDSP) overview (with link to EDSP assay validation)*. Washington, DC. https://www.epa.gov/endocrine‐disruption/endocrine‐disruptor‐screening‐program‐edsp‐overview

[bdr22075-bib-0053] U.S. Environmental Protection Agency (USEPA) . (2022). CompTox Chemicals Dashboard . https://comptox.epa.gov/dashboard/

[bdr22075-bib-0054] VanderMolen, K. M. , Naciff, J. M. , Kennedy, K. , Otto‐Bruc, A. , Shan, Y. , Wang, X. , … Mahony, C. (2020). Incorporation of in vitro techniques for botanicals dietary supplement safety assessment – Towards evaluation of developmental and reproductive toxicity (DART). Food and Chemical Toxicology, 144, 111539. 10.1016/j.fct.2020.111539 32645467

[bdr22075-bib-0055] Varghese, A. , Cawley, M. , & Hong, T. (2018). Supervised clustering for automated document classification and prioritization: A case study using toxicological abstracts. Environment Systems andDecisions, 38(3), 398–414. 10.1007/s10669-017-9670-5

[bdr22075-bib-0056] Wu, S. , Fisher, J. , Naciff, J. , Laufersweiler, M. , Lester, C. , Daston, G. , & Blackburn, K. (2013). Framework for identifying chemicals with structural features associated with the potential to act as developmental or reproductive toxicants. Chemical Research in Toxicology, 26(12), 1840–1861. 10.1021/tx400226u 24206190

