# Supplementary material for: Identifying the landscape of developmental toxicity new approach methodologies
Source: Birth Defects Res. 2022 Aug 12;114(17):1123–37. doi: 10.1002/bdr2.2075 (PMC9804744; doi:10.1002/bdr2.2075)
Supplement: Supplementary file 1 — Table S1 Search Term Combination Results Table S2. Topic Clustering Results [file BDR2-114-1123-s001.docx]

## Supplemental Material

### Annual Update Process

To ensure that the dashboard is representative of current DevTox NAMs research, a literature update will be conducted every year at least through 2023. The initial literature search was conducted in November of 2020, and the first literature update was conducted in August of 2021 using the same search strategy, the results of which are included in Figure 3 of the manuscript. While the literature update search strategy used was the same as the initial search, the prioritization and screening process was streamlined for efficiency and outlined below.

1. Topic clustering was not utilized in the update.
   1. The initial search used both topic clustering and supervised clustering to prioritize the studies. In the 2021 update only supervised clustering was utilized due to a lower number of references found during the search.
2. Supervised clustering approach was modified.
   1. The 2020 supervised clustering prioritized studies with a score of 4, 5, or 6 to move forward to screening phase. The 2021 update prioritized studies that scored a 5 or 6 due to the fact that cluster 4 only had a limited number of relevant results during the initial search.
   2. The 2020 supervised clustering used 25 references as seeds, while the 2021 update used those 25 plus an additional 25 relevant references identified during the 2020 initial search.
   3. We expanded supervised clustering to include the whole initial literature corpus during the update to capture any studies that may not have been captured during the first clustering effort utilizing additional relevant references as seeds. Through supervised clustering we were able to refine our seeds and continue to mine the literature corpus for any relevant studies
3. Title/Abstract Screening process was modified.
   1. To increase efficiency, of the approach of two screeners per reference and a third screener to resolving conflicts, the screening process was changed to one screener per reference.

### Table S-1: Search Term Combination Results

Various combinations of the strategy terms were tested and considered. The final chosen combination required terms from each of the four term sets and was selected as the strategy to be inclusive of the most relevant studies.

| Search | Description | PubMed |
| --- | --- | --- |
| 1 | Dev Tox AND (NAMs OR Models) | 373,390 |
| 2 | Dev Tox AND (NAMs OR Models)  Limited: English Only, 2010-present^a^ | 203,235 |
| 3 | Pathways AND (NAMs OR Models) | 128,717 |
| 4 | Pathways AND (NAMs OR Models)  Limited: English Only, 2010-present^a^ | 72,045 |
| 5 | Dev Tox AND Pathways AND (NAMs OR Models) | 128,717 |
| 6 | Dev Tox AND Pathways AND (NAMs OR Models)  Limited: English Only, 2010-present^a^ | 72,045 |
| 7 | Dev Tox AND NAMs AND Pathways AND Models | 17,192 |
| 8 | Dev Tox AND NAMs AND Models | 40,418 |
| 9 | Dev Tox AND NAMs AND Pathways | 119,208 |
| 10 | Dev Tox AND NAMs AND Pathways  Limited: English Only, 2010-present^a^ | 67,685 |
| 11 | NAMS AND Models AND Pathways | 67,685 |
| 12 | Dev Tox AND Models AND Pathways | 17,192 |

^a^ Present was defined as November 2020

### Table S-2: Topic Clustering Results

Search results were run through DoCTER’s Topic Extraction module. Topic Extraction is used to identify major themes in a body of literature by identifying common terms found in a reference’s title and abstract. The tool clusters the references into groups based on the shared terms. Topic Extraction results consist of a table which identifies the shared terms and the number of references that fall into each cluster; terms do not reflect the search strategy terms.

| Cluster | Count | Common Terms Identified |
| --- | --- | --- |
| 1 | 1218 | ['pluripotent stem', 'pluripotent', 'stem', 'human', 'stem cells', 'induced pluripotent', 'derived', 'induced', 'differentiation', 'human pluripotent', 'ipscs', 'stem cell', 'ipsc', 'disease', 'human induced', 'hpscs', 'vitro', 'neurons', 'culture', 'cell derived'] |
| 2^a^ | 2619 | ['drosophila', 'protein', 'gene', 'neurons', 'receptor', 'larval', 'function', 'genes', 'melanogaster', 'specific', 'proteins', 'neuronal', 'drosophila melanogaster', 'expressed', 'role', 'binding', 'nuclear', 'adult', 'developmental', 'brain'] |
| 3 | 722 | ['wnt', 'catenin', 'wnt signaling', 'beta catenin', 'signaling', 'beta', 'catenin signaling', 'pathway', 'canonical', 'canonical wnt', 'wnt catenin', 'xenopus', 'wnt pathway', 'tcf', 'wnt beta', 'axis', 'activation', 'embryos', 'signaling pathway', 'role'] |
| 4 | 2154 | ['stem', 'embryonic stem', 'stem cells', 'differentiation', 'embryonic', 'human', 'stem cell', 'es', 'human embryonic', 'mouse', 'derived', 'escs', 'es cells', 'pluripotency', 'vitro', 'hescs', 'pluripotent', 'hesc', 'signaling', 'mouse embryonic'] |
| 5 ^a^ | 711 | ['notch', 'notch signaling', 'signaling', 'pathway', 'delta', 'drosophila', 'notch pathway', 'fate', 'cell fate', 'notch signalling', 'differentiation', 'genes', 'role', 'signalling', 'function', 'signaling pathway', 'gene', 'activity', 'neural', 'specification'] |
| 6 | 3922 | ['zebrafish', 'embryos', 'xenopus', 'signaling', 'early', 'gene', 'protein', 'role', 'formation', 'function', 'embryonic', 'factor', 'genes', 'results', 'embryo', 'growth', 'model', 'expressed', 'activity', 'vertebrate'] |
| 7 ^a^ | 1251 | ['elegans', 'caenorhabditis', 'caenorhabditis elegans', 'daf', 'nematode', 'lin', 'pathway', 'dauer', 'protein', 'vulval', 'gene', 'signaling', 'genes', 'function', 'germline', 'developmental', 'mutants', 'genetic', 'proteins', 'nematode caenorhabditis'] |
| 8 | 552 | ['neural', 'crest', 'neural crest', 'zebrafish', 'crest cells', 'plate', 'craniofacial', 'xenopus', 'neural plate', 'signaling', 'migration', 'cranial', 'embryos', 'early', 'neural tube', 'nc', 'tube', 'genes', 'anterior', 'cranial neural'] |
| 9 | 1784 | ['zebrafish', 'exposure', 'toxicity', 'effects', 'embryos', 'exposed', 'induced', 'study', 'zebrafish embryos', 'developmental', 'model', 'apoptosis', 'thyroid', 'concentrations', 'genes', 'increased', 'larvae', 'receptor', 'used', 'using'] |
| 10 ^a^ | 2255 | ['drosophila', 'pathway', 'signaling', 'hh', 'eye', 'wing', 'hedgehog', 'required', 'dpp', 'function', 'gene', 'protein', 'polarity', 'patterning', 'genes', 'tissue', 'kinase', 'genetic', 'role', 'morphogenesis'] |

^a^Yellow Clusters were removed in an attempt to reduce the number of genomic studies and discovery research into gene structure and function because the target was to identify applied methodologies for NAMs that could be advanced for developmental toxicity screening.
